# Combining Missing Data Imputation and Internal Validation in Clinical Risk Prediction Models

**DOI:** 10.1002/sim.70203

**Published:** 2025-08-07

**Authors:** Junhui Mi, Rahul D. Tendulkar, Sarah M. C. Sittenfeld, Sujata Patil, Emily C. Zabor

**Affiliations:** ^1^ Department of Quantitative Health Sciences Cleveland Clinic Research Cleveland Ohio USA; ^2^ Department of Radiation Oncology Taussig Cancer Institute, Cleveland Clinic Cleveland Ohio USA; ^3^ Department of Radiation Oncology The Barrett Cancer Center, University of Cincinnati Cincinnati Ohio USA

**Keywords:** deterministic imputation, imputation, missing data, multiple imputation, prediction model, risk prediction

## Abstract

Methods to handle missing data have been extensively explored in the context of estimation and descriptive studies, with multiple imputation being the most widely used method in clinical research. However, in the context of clinical risk prediction models, where the goal is often to achieve high prediction accuracy and to make predictions for future patients, there are different considerations regarding the handling of missing covariate data. As a result, deterministic imputation is better suited to the setting of clinical risk prediction models, since the outcome is not included in the imputation model and the imputation method can be easily applied to future patients. In this paper, we provide a tutorial demonstrating how to conduct bootstrapping followed by deterministic imputation of missing covariate data to construct and internally validate the performance of a clinical risk prediction model in the presence of missing data. Simulation study results are provided to help guide when imputation may be appropriate in real‐world applications.

**Abbreviations**
ANA: anti‐nuclear antibodies
APC: antigen‐presenting cells
IRF: interferon regulatory factor

## 1 Introduction

Clinical risk prediction models are widely used in medical settings to predict specific outcomes for individual patients. These models generally fall into two categories: diagnostic models, which aim to predict whether or not a patient has a certain disease or condition, and prognostic models, which aim to predict whether or not a patient will develop a certain outcome in the future. This paper will focus on prognostic clinical prediction models. There are three stages in the creation of a clinical risk prediction model: development, validation, and deployment. Development describes the process of determining what variables will be used and building an appropriate model to predict the outcome of interest. Validation refers to the step of using a separate dataset from what was used in the development stage to test the performance of the model on an independent dataset. Finally, deployment is the stage when the model is made available either online or in a publication so that it can be applied to data from a new patient to generate a predicted risk of the patient experiencing the outcome of interest. It is possible to encounter missing data across all three stages in the generation of a new prediction model. One approach to handle missing data is imputation, where the missing value is replaced by a substitute value. Employing imputation in the context of a clinical risk prediction model requires careful thought about all three stages and an understanding of when it is appropriate to employ imputation. In this paper, we provide a tutorial demonstrating how to conduct imputation in the development and validation processes in a way that simplifies the deployment of a clinical risk prediction model.

Missing data can arise for many different reasons. In one setting, most variables are complete, but one variable of interest is missing for some percentage of patients. This could be a self‐reported variable like race, which may be absent if patients decline to answer, or it could be a costly biomarker that is not tested on a large percentage of patients. In another setting, a number of variables are missing to various degrees for some patients, which is common in electronic health records, where different practitioners may routinely enter different information for their patients. In a third setting, some patients have complete data, but other patients are missing multiple variables, which may arise due to differences in insurance, provider practices, or access to care, among other reasons. There is currently a lack of guidance about when it is appropriate to apply imputation in the context of building a clinical risk prediction model. To address this, this paper also describes results from a simulation study to help guide practitioners on when it may be acceptable to employ imputation when building a clinical risk prediction model in the presence of missing data.

The rest of the paper is arranged as follows. Sections 2 and 3 provide more in‐depth background on approaches to missing data imputation and validation of clinical risk prediction models, respectively. Section 4 describes the metrics of predictive performance that will be used in this paper to evaluate the risk prediction models. Section 5 describes the motivating clinical example and the procedures used to generate synthetic data, impose missingness, and impute missing values. In Section 6, we walk through a guided data analysis example, including detailed code in the R [1] programming language. Section 7 describes the simulation study design and results. Finally, we conclude with a summary and discussion in Section 8.

## 2 Imputation Methods

Multiple imputation has been the gold standard to handle missing data in clinical research for some time now [2]. Multiple imputation is a form of stochastic imputation, which refers to an approach grounded in Bayesian statistical theory where missing values are sampled from a distribution of possible values. The “multiple” part of multiple imputation indicates that the process of drawing values is repeated several times, resulting in multiple datasets that each contain different imputed values for the same individual missing value [2]. Stochastic imputation methods, such as multiple imputation, are focused on adjusting the variability of estimates to account for the uncertainty in the imputation modeling process. When the imputation model is properly specified, multiple imputation results in valid inference for regression coefficients that result from models that are fit to the multiply imputed datasets and then combined in an appropriate fashion to account for both within and between imputation uncertainty [2, 3, 4].

Deterministic imputation is an alternative to multiple imputation. Also known as regression imputation, deterministic imputation fits a single imputation model and then directly replaces the missing value in a single dataset with a single fixed predicted value. There is no implicit consideration of the imputation error, but in certain settings it is possible to account for the imputation error after the fact using a resampling approach such as bootstrapping, as long as the entire fitting procedure from the imputation model through the estimation model is included in the resampling procedure [5].

In the context of a clinical prediction model, missing data needs to be considered separately across the three stages of development, validation, and deployment. Missing data on predictors may be present at every step. For the prognostic prediction models under consideration here, the outcome will always be unknown at the time of model deployment, when it is of interest to use a new patient's covariate values, some of which may be missing, to predict a future outcome that has not yet occurred and is therefore missing by design. A common recommendation is to include the outcome in the imputation model to achieve unbiased results. But this recommendation is actually specific to stochastic imputation, for which it is a requirement rather than a recommendation [5]. It has been mathematically proven that the outcome *must* be included in the imputation model to ensure unbiased results from stochastic imputation, whereas the outcome *must not* be included in the imputation model to ensure unbiased results from deterministic imputation [5]. Previous simulation studies have shown that when the outcome is appropriately omitted from the imputation model, deterministic imputation has comparable performance to multiple imputation on metrics relevant to clinical risk prediction models such as discrimination, accuracy, and calibration [6].

An additional consideration at the time of model deployment is computational efficiency. Deterministic imputation is straightforward and computationally fast to apply to future patients. It first uses the static imputation model to generate fixed imputed values. Then, it applies the static prediction model to the combination of known and imputed covariate values and generates a predicted outcome. The development data are not required for this process, nor is the new patient's unknown outcome value. In contrast, multiple imputation approaches require access to the full development dataset, and new computations are necessary to obtain a prediction for a future patient [7]. The standard approach is to stack the new patient's data onto the development data prior to refitting the imputation models, generating the multiply imputed datasets, fitting the regression models for the outcome, and combining the resulting multiple estimates. In the context of a prognostic prediction model for which the new patient's outcome is unknown at the time of prediction, multiple imputation would first impute the outcome value prior to imputing any missing covariate values, before finally using the imputed covariates to then predict the outcome value [6]. This process is computationally intensive, may take some time to produce a result, and would require secure storage of the development data.

For these reasons, when the goal is to deploy a publicly available prognostic risk prediction model that will be used to predict the unobserved outcome for a new patient's data, deterministic imputation may be preferable to multiple imputation.

## 3 Validation Techniques

After model development and prior to deployment, clinical prediction models require validation. External validation refers to the setting where completely separate datasets are used for model development and validation. For example, data from one hospital may be used for model development, and data from another hospital may be used for model validation. Or data from one time period may be used for model development, and data from a later time period may be used for model validation. While external model validation has often been considered the gold standard for performance assessment of clinical risk prediction models, it is common in practice to only have access to a single dataset during the model‐building process, so that internal validation is required. Internal validation refers to the setting where a single dataset is used for both model development and validation. To accomplish both, the dataset must be split in some way. Common internal validation procedures include a single random split, cross‐validation, and bootstrapping. Unless an unusually large dataset is available at the time of model development, the tradeoff of reduced sample size to achieve a single random split is not beneficial [8]. In this context, a resampling procedure such as cross‐validation or bootstrapping is recommended. The resampling procedure should encompass the entire model‐building process, including any variable selection or model selection, as well as any imputation procedure. When internal validation is done with bootstrapping, it can even outperform external validation in some settings [8].

Because it is more common to only have access to a single dataset for model development and validation, we will focus here on conducting internal validation with bootstrapping. When the single dataset contains missing predictor data, we will need to combine the imputation and bootstrapping procedures. Now we may wonder: in which order should we perform the bootstrapping and imputation? Should we impute the missing predictor values first, and then bootstrap the complete imputed data? Or should we bootstrap the incomplete original data, and separately impute missing predictor values into each bootstrap sample? In the setting where estimation is of interest, it has been shown that bootstrapping prior to multiple imputation achieves superior confidence interval coverage [9, 10]. When prediction is of interest, we would argue that bootstrapping prior to imputation is the only acceptable approach, since doing imputation prior to bootstrapping could be considered using information from the development process in the validation process. Therefore, we recommend performing bootstrapping prior to imputation.

## 4 Metrics of Predictive Performance

This paper is focused on the specific setting where the goal is to develop and validate a clinical prediction model to be employed to predict a future patient's outcome, when there is a single dataset available for both development and validation, and there may be missing predictor variables. Given this context, we evaluated the performance of the described methods using traditional performance measures for clinical prediction models, incorporating resampling methods to correct for overfitting and obtain more robust performance estimates. Specifically, we assessed the model's discriminative ability using the area under the receiver operating characteristic curve (AUC) and measured overall model performance with the Brier score [11] and assessed calibration by evaluating individual patient predicted values. Individual patient predicted values were used to assess calibration in this case, as the ultimate goal of a clinical prediction model is to predict the future outcome for an individual new patient. The AUC represents the probability that a randomly chosen positive instance (event) ranks higher in predicted risk than a randomly chosen negative instance (non‐event), with an AUC of 1 indicating perfect discrimination [12]. For time‐to‐event outcomes, the AUC is adapted to a time‐dependent AUC, which evaluates the model's discriminative capacity at specific time points. The Brier score assesses the accuracy of risk predictions by calculating the mean squared difference between predicted probabilities and observed outcomes, where a Brier score of 0 indicates perfect accuracy [12]. For survival analysis, the time‐dependent Brier score is used, calculating the difference between predicted survival probabilities and actual outcomes at specific time points. We reported both apparent (uncorrected) AUC and Brier scores and bias‐corrected estimators, as detailed below.

### 4.1 Apparent

The apparent estimator measures predictive performance directly on the original sample, often yielding overly optimistic results as the model is evaluated on the same data used for its development. This can lead to an underestimation of the true prediction error [13].

### 4.2 Bootstrap‐Corrected Estimator

The bootstrap‐corrected estimator, introduced by Harrell [14] as a bias correction estimator, adjusts the apparent estimator by accounting for optimism, defined as the average difference between the performance on bootstrap samples and the original dataset. By subtracting this optimism, the corrected estimator provides a more realistic assessment of model performance, accounting for overfit. However, because approximately 63.2% of the original sample is typically included in each bootstrap sample, this overlap can still lead to a slight overestimation of performance [13].

### 4.3 0.632 Estimator

The 0.632 bootstrap method addresses the potential optimistic bias of conventional bootstrap validation by weighting performance on in‐sample data at 0.632 and out‐of‐sample, or test, data at 0.368 [15]. This weighting can produce a more balanced estimate.

### 4.4 0.632+ Estimator

As a modification of the 0.632 method, the 0.632+ estimator further adjusts for overfit models by dynamically changing the weights based on an estimate of the amount of overfit in the model. [16] This approach is particularly suitable for complex models, as it optimally balances in‐sample and out‐of‐sample performance, making it ideal for high‐risk overfitting scenarios. When overfitting is minimal, the 0.632+ and 0.632 estimators yield similar results. Calculating the 0.632+ estimator requires selecting a benchmark value representing a non‐informative model. For the AUC, this value is 0.5. For the Brier score, a value of 0.25 represents a non‐informative model where one predicts a 50% risk for all participants [11, 17].

## 5 Data Generation

### 5.1 Motivating Dataset

This study is motivated by a case study where interest was in the association between post‐mastectomy radiation therapy (PMRT) and breast cancer outcomes, adjusted for a variety of other patient and disease characteristics [18]. In the original study, there was interest in both estimation and prediction. Eleven covariates were selected a priori for inclusion in the prediction model. The primary predictor of interest, PMRT, had no missing data. Six of the other covariates had missing data, with four missing in <3% of patients and two missing in >15% of patients. Seventy‐eight percent of patients had complete covariate data, 21% were missing one covariate, 1.2% were missing two covariates, and 0.03%(n=1) were missing three covariates. The original study employed multiple imputation of missing data values followed by bootstrapping for interval validation. The dataset consisted of 3532 patients, of whom 2750 had complete data. Median follow‐up time among survivors was 6.8 years (Range: 0.4–13.8). During that time, 626 patients died from any cause.

### 5.2 Synthetic Data Generation

Simulated data will be generated for use throughout this paper based on the observed distributions of covariates and associations between covariates and outcome in the previously described motivating dataset [18]. While the original study investigated a variety of clinical outcomes, some in the context of competing risks, here the focus is only on associations with overall survival. Overall survival is a right‐censored time‐to‐event outcome, with time measured from the date of diagnosis to the date of death. Patients who have not yet died are censored at the last date they were known to be alive.

Death and censoring times were generated using the Weibull distribution. The baseline shape and scale parameters for the distribution of death times were estimated using a parametric Weibull model fit to the complete case dataset, including all 11 covariates, resulting in a shape parameter of 1.6 and a scale parameter of 122. The baseline shape and scale parameters for the distribution of censoring times were estimated using a parametric Weibull model fit to the full dataset with no covariates, resulting in a shape parameter of 2.6 and a scale parameter of 8.2. True death times were generated from a Weibull distribution using the inverse uniform transform method. First, a uniform random variable, u, was generated. Then, Weibull death times were calculated as $t_{i}=b\times {\left((-\log(u_{i}))/\exp(\beta^{\prime }X_{i})\right)}^{\left(1/a\right)}$, where a is the Weibull shape parameter, b is the Weibull scale, $\beta^{\prime }$ is the vector of coefficients for the covariates, $X_{i}$ is the vector of covariate values, $\left(X_{i1},\ldots ,X_{ip}\right)$, for covariate j=1,…,p and participant i=1,…,n. Censoring times $c_{i}$ were simulated directly from a Weibull distribution. Observed overall survival time for each participant is $s_{i}=\min(t_{i},c_{i})$. The event, $\delta_{i}$, is death (1) if $t_{i}\leq c_{i}$ and censored (0) if $t_{i}>c_{i}$.

The true hazard ratios and type for each covariate are shown in Table 1. Covariate values were generated from a multivariate normal distribution, using mean vector (0.6145,57.6495,2.3665,0.4225,0.1538,0.2421,0.4142,0.8680,0.1695,0.1636,0.8891) for $\left(X_{1},\ldots ,X_{11}\right)$, respectively, and the covariance matrix shown in Table 2, both estimated from the original dataset. Binary variables were dichotomized at 0.5.

**TABLE 1:** True hazard ratios.

Covariate	Type	Hazard ratio
$X_{1}$	Binary	0.80
$X_{2}$	Continuous	1.05
$X_{3}$	Continuous	1.25
$X_{4}$	Binary	1.54
$X_{5}$	Binary	1.18
$X_{6}$	Continuous	1.45
$X_{7}$	Binary	1.10
$X_{8}$	Binary	0.76
$X_{9}$	Binary	0.64
$X_{10}$	Binary	1.25
$X_{11}$	Binary	0.48

**TABLE 2:** Covariance matrix for synthetic data.

	$X_{1}$	$X_{2}$	$X_{3}$	$X_{4}$	$X_{5}$	$X_{6}$	$X_{7}$	$X_{8}$	$X_{9}$	$X_{10}$	$X_{11}$
$X_{1}$	0.2370										
$X_{2}$	−1.3349	196.2990									
$X_{3}$	0.0812	0.6471	1.0967								
$X_{4}$	0.0247	−0.6314	0.0680	0.2441							
$X_{5}$	0.0298	−0.0759	0.0452	0.0063	0.1302						
$X_{6}$	0.0134	0.3167	0.0063	−0.0089	0.0070	0.0558					
$X_{7}$	0.0280	−0.7944	0.0740	0.0468	0.0214	−0.0015	0.2427				
$X_{8}$	−0.0069	0.0156	−0.0190	−0.0581	−0.0026	0.0050	−0.0119	0.1146			
$X_{9}$	0.0039	−0.1261	−0.0080	0.0484	0.0016	−0.0026	0.0131	−0.0318	0.1408		
$X_{10}$	0.0002	0.0294	−0.0147	0.0003	−0.0001	0.0003	0.0017	0.0001	0.0050	0.1369	
$X_{11}$	0.0223	−0.8057	−0.0075	−0.0139	0.0043	−0.0000	0.0012	0.0086	−0.0267	0.0014	0.0986

### 5.3 Missing Data Generation

Missingness was imposed using a missing at random (MAR) framework, where missingness of a given covariate may depend on observed values but occurs independently of any unobserved values, following the approach of Marshall et al. [19] and Wahl et al. [12]. Let $X_{ij}$ denote the jth covariate for observation i, with i=1,…,n and j=1,…,p. $M_{ij}$ denotes the indicator for missingness, $M_{ij}=I(X_{ij}\;\text{missing})$. Then, the probability of missingness for each covariate value was modeled as a function of the value of one other covariate and the missingness status of one other covariate as

$$P(M_{ij}=1)=\text{logistic}(\gamma_{0j}+\gamma_{1j}M_{ik_{j}}+\gamma_{2j}X_{il_{j}})$$

where $M_{ik_{j}}$ denotes the missingness of a randomly chosen covariate and $X_{il_{j}}$ denotes the observed value of a randomly chosen other covariate, such that $k_{j}\neq l_{j}$.

$\gamma_{1j}$ was defined as

$$\gamma_{1j}=\left\{\begin{array}{ll} 0,\; & \text{if}\;j=1\\ \log(OR_{jk_{j}}),\; & \text{otherwise} \end{array}\right.$$

$OR_{jk_{j}}$ was calculated based on a cross‐tabulation of the missing proportions for $X_{ij}$ and $X_{ik_{j}}$. Let $p_{rc}$ denote the cell proportion of missingness for the cross tabulation of $M_{ij}$ in the row so that r takes the value 0 if $M_{ij}$ is not missing and the value of 1 if $X_{ij}$ is missing, and $X_{ik_{j}}$ in the column so that c takes the value of 0 if $X_{ik_{j}}$ is not missing and the value of 1 if $X_{ik_{j}}$ is missing. Then $OR_{jk_{j}}=\frac{p_{00}\times p_{11}}{p_{10}\times p_{01}}$.

The intercepts $\gamma_{0j}$ were estimated by solving the equation

$$\gamma_{0j}=\log\left(\frac{P(M_{ij}=1)}{1-P(M_{ij}=1)}\right)-\gamma_{1j}P(M_{ik_{j}}=1)-\gamma_{2j}{\bar{X}}_{il_{j}}$$

The specific missing data mechanisms for each covariate with potential missingness, and the fixed values of $\gamma_{2j}$, are shown in Table 3.

**TABLE 3:** Fixed probability of missingness parameters.

Missing covariate	$k_{j}$	$l_{j}$	$\gamma_{2j}$
$X_{1}$	—	2	log(1.05)
$X_{3}$	1	5	log(0.80)
$X_{4}$	3	6	log(0.70)
$X_{7}$	4	8	log(0.90)
$X_{10}$	5	9	log(0.60)
$X_{11}$	10	2	log(1.05)

### 5.4 Missing Data Imputation

The deterministic regression imputation method uses a set of complete covariates to generate a single predicted value for each missing covariate value [20, 21]. In deterministic regression imputation involving multiple missing variables, there are two key strategies: using separate imputation models for each variable or employing a sequential imputation approach. In our analysis, we employed deterministic regression imputation using independent models for each variable. Both sequential imputation and independent imputation are valid approaches, and the choice between them depends on the missing data structure, inter‐variable relationships, and the objectives of the analysis [20, 22]. For each missing variable, a generalized linear model was fit with the missing variable as the outcome and all other complete variables as the predictors, in the subset of patients not missing the outcome variable. The link function was logit for binary missing variables and identity for continuous missing variables. Next, predicted response values for the missing variable were generated to serve as imputed values.

## 6 Guided Example

The guided example is meant to mirror the setting where we have a single clinical dataset that contains missing values for some predictors, and the goal is to construct and internally validate a clinical risk prediction model. In this setting, the “truth”, based on full data with no missingness, is unknown. The guided example considers the setting with missingness in three covariates $X_{1}$, $X_{3}$, and $X_{4}$ such that $P(M_{i1}=1)=0.05$, $P(M_{i3}=1)=0.15$, and $P(M_{i4}=1)=0.30$. The joint missingness proportions were $P(M_{i1}=1\;\text{and}\;M_{i3}=1)=0.02$ and $P(M_{i3}=1\;\text{and}\;M_{i4}=1)=0.075$. The other eight covariates are complete. In line with the motivating example, the outcome of interest is a time‐to‐event outcome, and the data are analyzed using multivariable Cox regression. The R code to generate and analyze the single synthetic dataset used in this section, as well as the data file itself, is available on GitHub at https://github.com/zabore/manuscript‐code‐repository/tree/master/Mi‐Zabor_bootstrap‐impute‐predict‐tutorial. Before running the code in this section, either load the data file or run the code to generate the dataset to have access to the data frame object dat0 used in the guided example. Here, the prediction horizon is set to 5 years, and 500 bootstrap samples were generated.

First, load the R packages needed for this analysis [1, 17, 23, 24, 25, 26, 27].

 

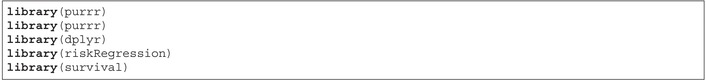

To perform imputation on both the original dataset and each bootstrap sample separately, we incorporate the imputation code within a function. The first step fits the imputation models with each variable with missing data as the outcome, in the subset of patients not missing that variable. The second step generates a predicted value for each variable with missing data for all patients in the dataset, in line with the first imputation method described in Section 5.4. The final step keeps the predicted value for patients missing the variable of interest and keeps the original value for patients not missing the variable of interest.

 

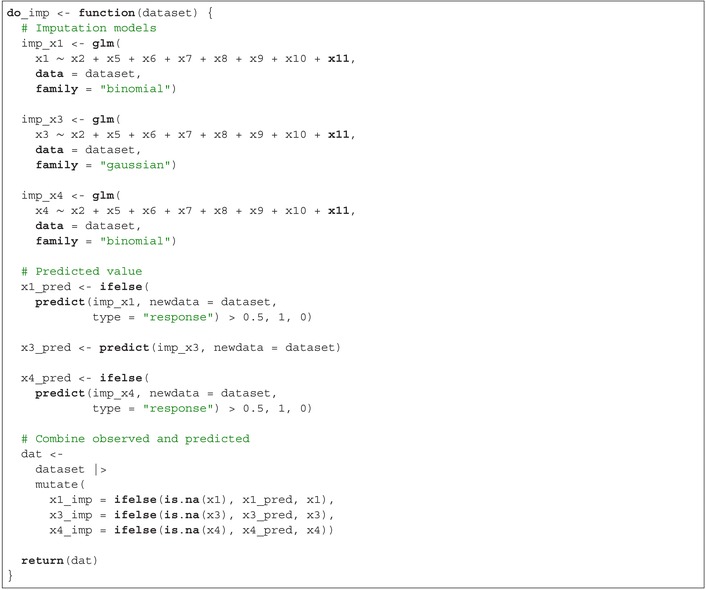

Use the do_imp() function to impute directly into the original dataset dat0, creating a new data frame object dat:

 

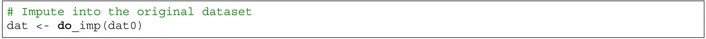

Next, generate 500 bootstrap samples with replacement, and impute into each bootstrap sample:

 

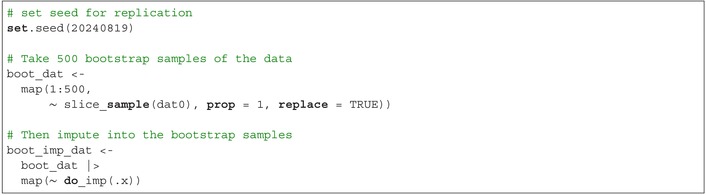

Determine the apparent performance by fitting a multivariable Cox model to the original imputed dataset and evaluating its performance through AUC and Brier score:

 

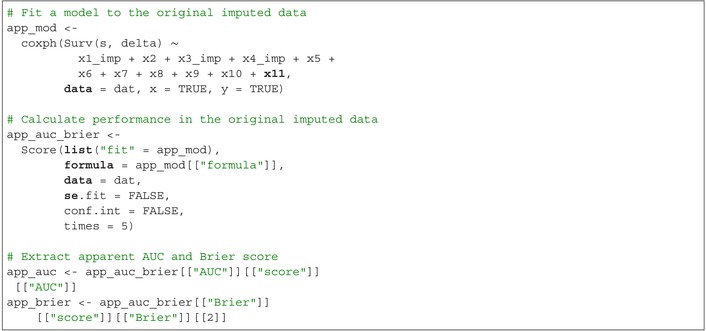

The apparent AUC is app_auc=0.722 and the apparent Brier score is app_brier=0.092. Then, calculate Harrell's bootstrap‐corrected performance by fitting a multivariable Cox regression model to each bootstrap sample and evaluating its performance in both the bootstrap sample and the original imputed dataset. Harrell's bootstrap‐corrected performance is calculated as the apparent performance minus the average difference between the performance in each bootstrap sample and the performance in the original imputed data:

 

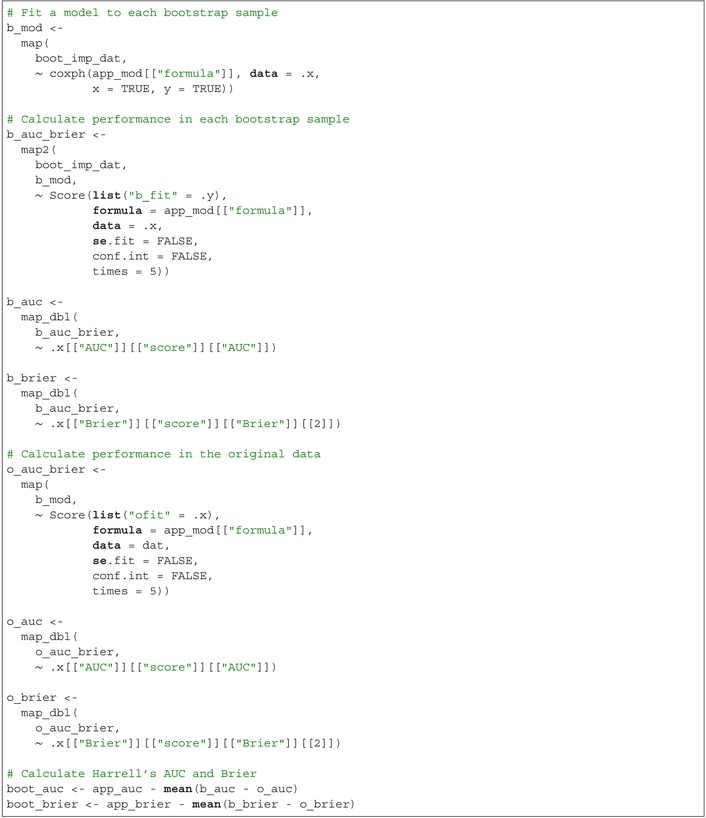

Harrell's bootstrap‐corrected AUC is boot_auc=0.717 and Harrell's bootstrap‐corrected Brier score is boot_brier=0.093. Next, calculate the 0.632 performance. Begin by creating test datasets for each bootstrap sample, consisting of records from the original imputed dataset that were not included in the respective bootstrap sample. Then, evaluate the performance of the models fitted on each bootstrap sample using their corresponding test datasets. The 0.632 performance is calculated as 0.368 times the apparent performance plus 0.632 times the average performance across the test datasets:

 

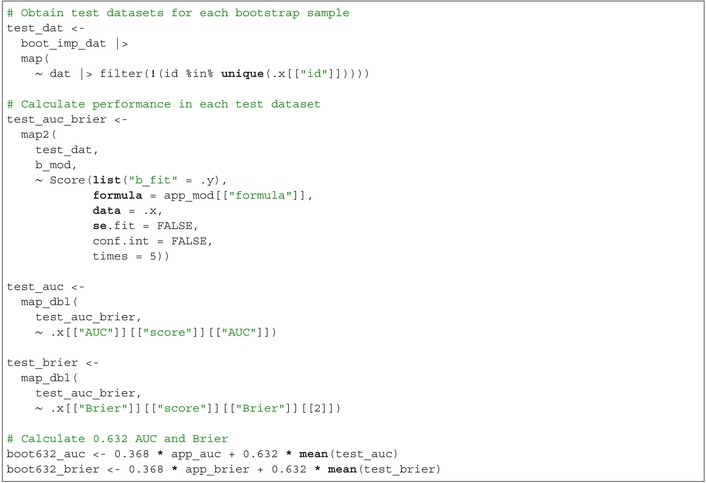

The 0.632 AUC is boot632_auc=0.717 and the 0.632 Brier score is boot632_brier=0.093. Finally, calculate the 0.632+ performance. First, fix the “no information performance” at 0.5 for the AUC and 0.25 for the Brier score, representing the value of the performance metric for a non‐informative predictive model. The relative overfitting rate is calculated as the difference between the average performance in the test datasets minus the apparent performance, divided by the difference between the “no information performance” value and the apparent performance. Next, define weights as 0.632 divided by 1 minus 0.368 times the relative overfitting rate. Finally, the 0.632+ performance is calculated as 1 minus the weight times the apparent performance plus the weight times the average performance in the test datasets:

 

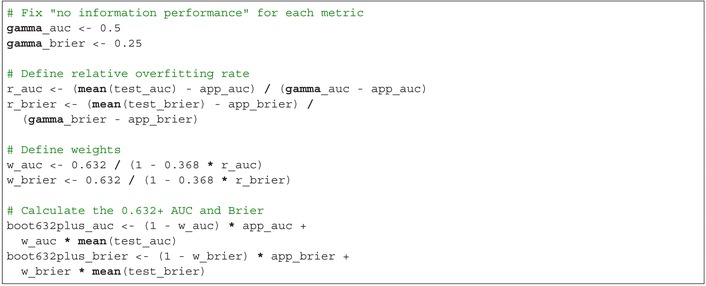

The 0.632+ AUC is boot632plus_auc=0.717 and the 0.632+ Brier score is boot632plus_brier = 0.093.

From the guided example, we find that our original model was not very overfit, as the difference between the apparent performance metrics and Harrell's bootstrap‐corrected performance metrics is somewhat small. We also had a little overestimation of performance based on the inclusion of, on average, 63.2% of patients from the original data in each bootstrap sample, as reflected in the similarly small difference between Harrell's bootstrap‐corrected performance metrics and the 0.632 performance metrics. The 0.632+ performance metrics are identical to the 0.632 performance metrics, which is expected in this setting, given the lack of strong overfit in the original model.

## 7 Simulation Study

We now describe a simulation study to evaluate the performance of different imputation strategies in estimating predictive performance metrics from a survival model. Specifically, it assesses how different strategies influence the accuracy of the AUC, the Brier score, and individual‐level predicted probabilities under various missing data scenarios.

### 7.1 Design of Simulation Study

This simulation study aims to evaluate the bias, efficiency, and coverage probability of the AUC as a measure of model discrimination and the Brier score as a measure of overall accuracy, as detailed in Section 4, and the bias of individual patient predicted values.

Data for the simulation study were generated from a parametric model with fixed parameters that were based on the real data example, as detailed in Section 5.2. The simulation study evaluated 54 scenarios in a factorial manner, considering two sample sizes (750, 3500), nine missing data patterns, and three imputation methods. The scenarios are detailed in the Appendix: Table A1. The three imputation strategies were: (1) imputing all missing values, (2) imputing only for variables missing across >10% of participants, and (3) imputing only for participants missing two or fewer variables. The nine missing data patterns included: $x_{1}$ missing at (A) 5%, (B) 15%, (C) 60%; $\left(x_{1},x_{3},x_{4}\right)$ missing at (D) (5%, 5%, 5%), (E) (5%, 15%, 30%), and (F) (15%, 30%, 60%); and $\left(x_{1},x_{3},x_{4},x_{7},x_{10},x_{11}\right)$ missing at (G) (5%, 5%, 5%, 5%, 5%, 5%), (H) (5%, 5%, 15%, 15%, 30%, 30%), and (I) (15%, 15%, 30%, 30%, 60%, 60%). To reflect the complex joint missingness seen in real‐world data, we applied pre‐specified marginal and joint missingness proportions (Table 4), following the specific missing data mechanisms for each covariate as outlined in Table 3.

**TABLE 4:** Marginal and joint missing data proportions.

Marginal missingness $X_{i}$	Marginal missingness $X_{j}$	Joint missingness $\left(X_{i},X_{j}\right)$
0.05	0.05	0.01
0.05	0.15	0.02
0.15	0.15	0.05
0.15	0.30	0.07
0.30	0.30	0.10
0.30	0.60	0.20
0.60	0.60	0.40

The primary estimands were the time‐dependent AUC and Brier score at 5 years, along with the average predicted survival probabilities at 5 years for each individual. These were chosen to reflect both population‐level and individual‐level model performance in the presence of missing covariate data.

For each approach, we applied 500 bootstrap replications to account for sampling variability, followed by deterministic regression imputation (BI). Complete case analysis (CC) was also conducted as a reference. The bias of the AUC and Brier score was calculated as the difference between the performance metric computed on the full data and the performance metric employing CC and BI approaches. The bias of individual patient predicted probabilities was calculated as the difference in the average predicted probability in the full data and the average predicted probability employing CC and BI approaches, applying the appropriate complementary log‐log and inverse complementary log‐log transformations. A total of 1000 simulated datasets were generated.

### 7.2 Results of Simulation Study

**FIGURE 1:**
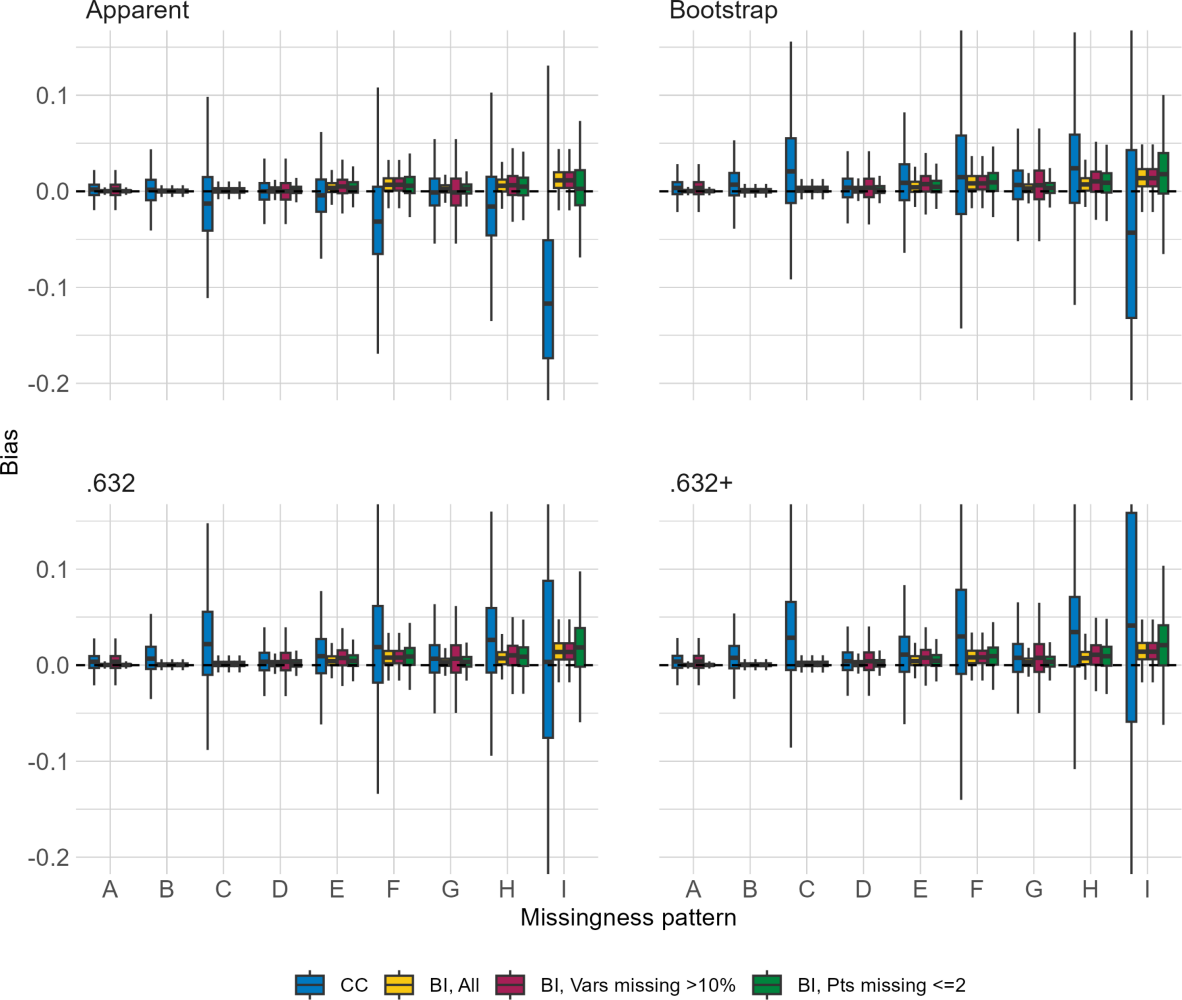
Distribution of bias (y‐axis) of AUC estimators (panels) comparing the CC approach and the BI approach with three imputation methods (color) when the sample size is 750 across missing data patterns (x‐axis).

**FIGURE 2:**
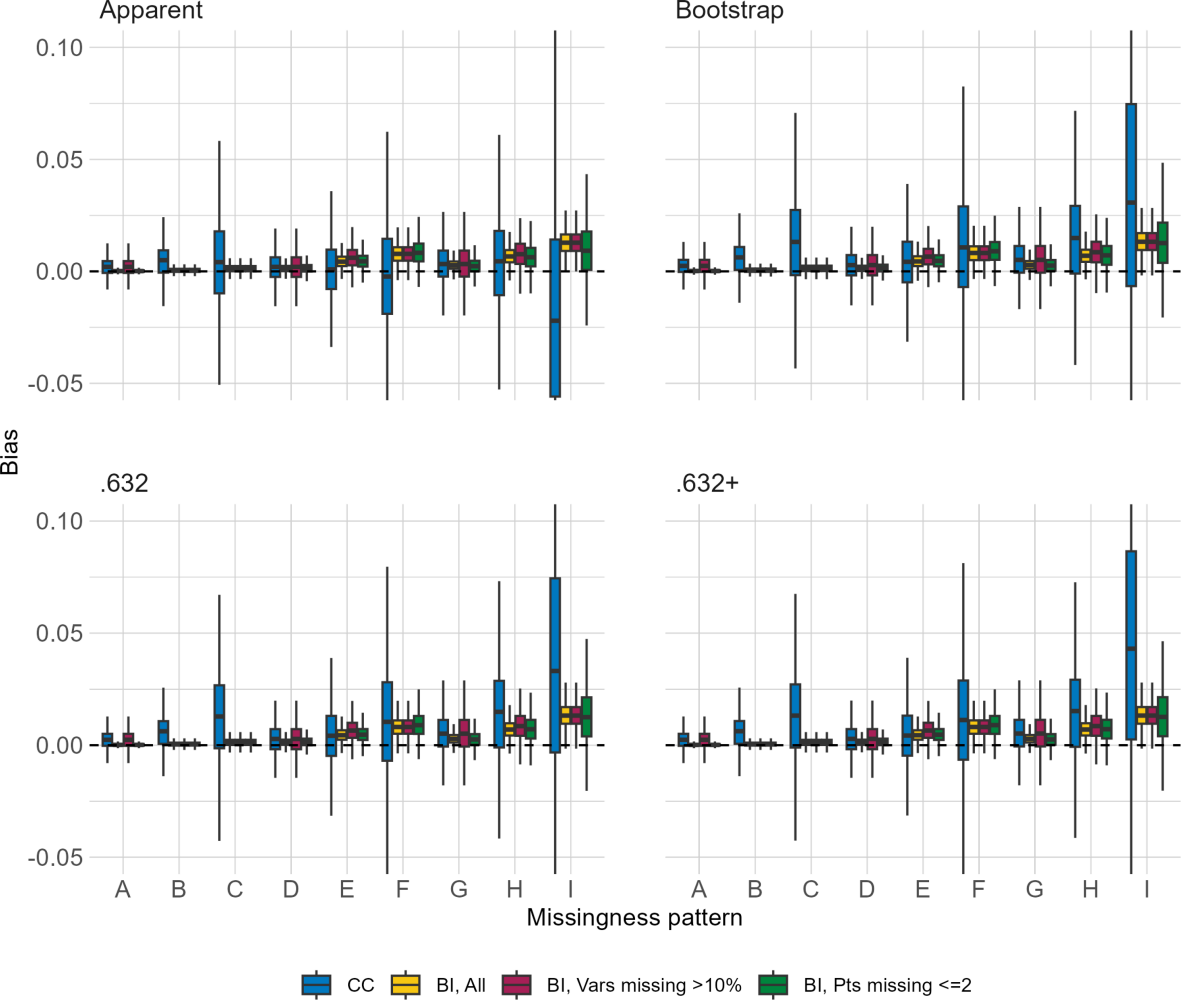
Distribution of bias (y‐axis) of AUC estimators (panels) comparing the CC approach and the BI approach with three imputation methods (color) when the sample size is 3 500 across missing data patterns (x‐axis).

**FIGURE 3:**
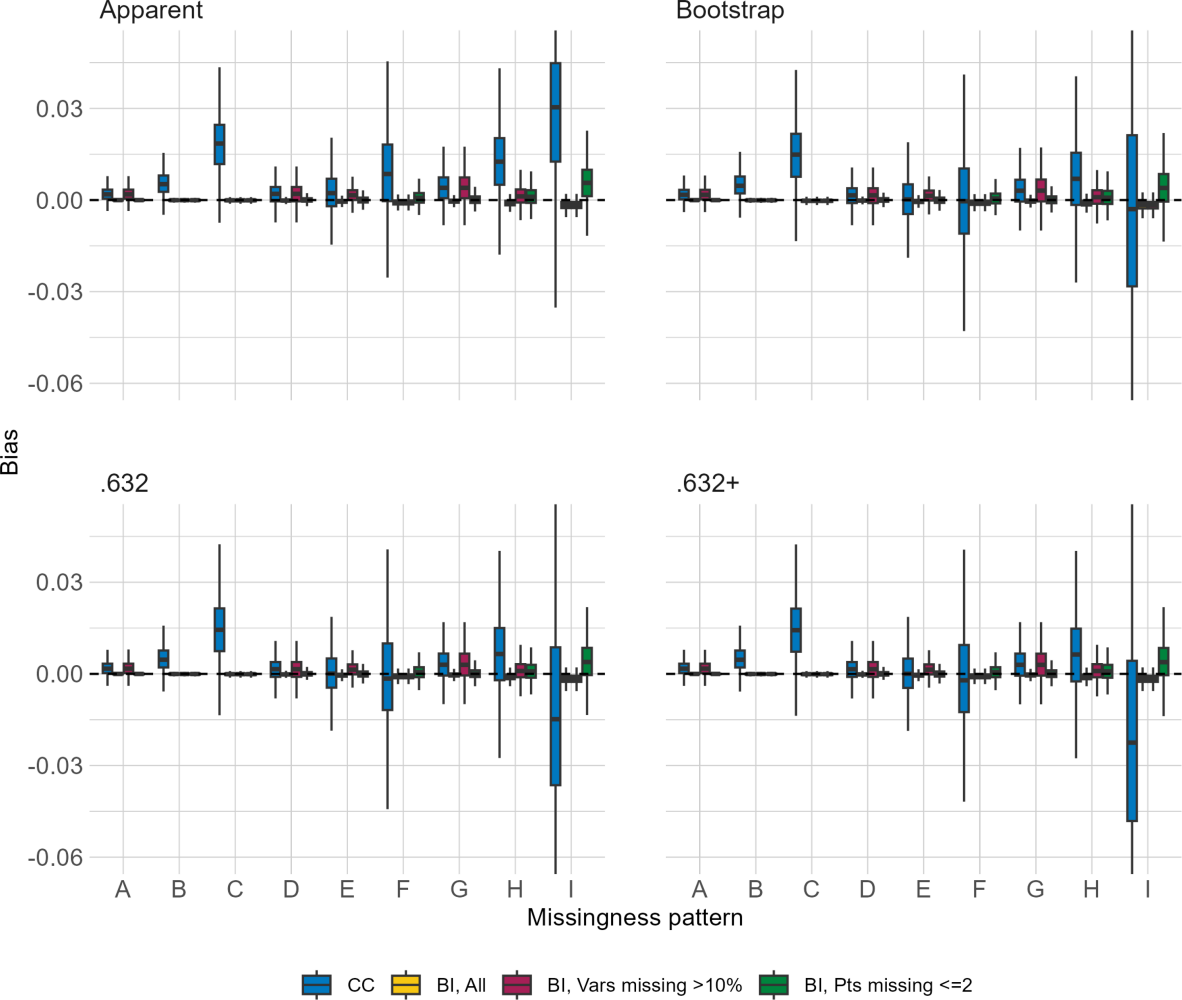
Distribution of bias (y‐axis) of Brier score estimators (panels) comparing the CC approach and the BI approach with three imputation methods (color) when the sample size is 750 across missing data patterns (x‐axis).

**FIGURE 4:**
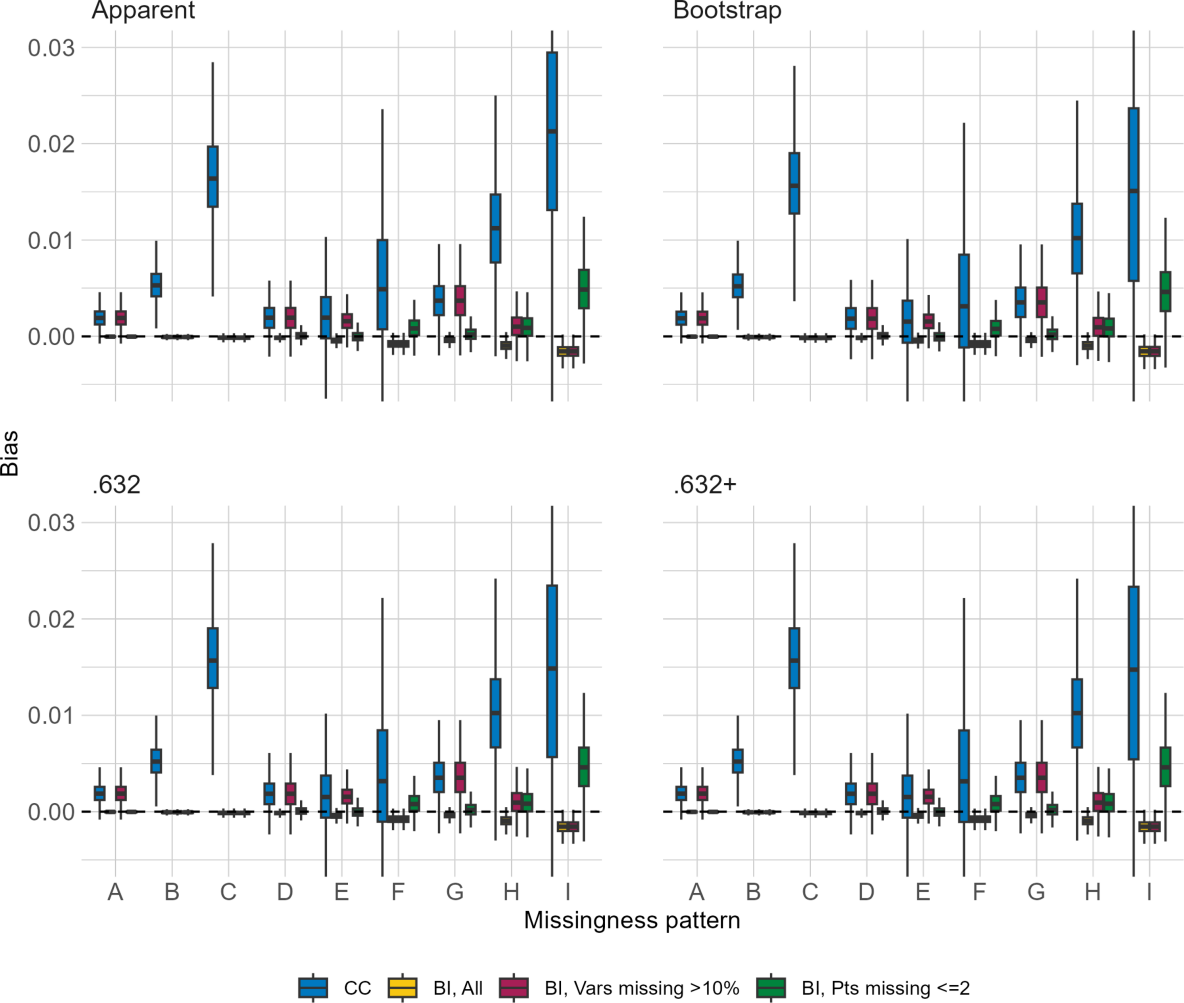
Distribution of bias (y‐axis) of Brier score estimators (panels) comparing the CC approach and the BI approach with three imputation methods (color) when the sample size is 3 500 across missing data patterns (x‐axis).

The distributions of bias across the nine missing data patterns for each of the four performance metrics are shown in Figures 1, 2, 3, 4. Each figure presents results for a combination of a single sample size for either the AUC or the Brier score. When the sample size is 750 and the approach is CC, the complete data were too sparse to successfully fit the multivariable model of interest in 0.3%, 1.2%, 0.4%, and 57.7% of simulated datasets for missing data patterns C, F, H, and I, respectively. These are missing data patterns with either 60% missingness for a given covariate or with missingness in 6 covariates, some of which are missing in >5% of participants. Even when the sample size is 3500, the CC approach is unable to fit the multivariable model of interest for missing pattern I in 0.3% of simulated datasets. The figures are based on results from simulated datasets where the multivariable model of interest was successfully fit, resulting in smaller sample sizes for certain settings.

The CC approach always has more bias and more variability in the bias, as compared to the BI approach with any of the imputation methods. For the BI approach, the bias is equal or lower across almost all settings when we impute all versus only imputing covariates missing >10% or only imputing for participants with ≤2 missing covariates, the approach with the highest bias. This holds true even for missing data patterns with higher levels of missingness within a covariate or higher numbers of missing covariates. With respect to the missing data pattern, the bias is larger for the more extreme missing data patterns. With the CC approach, regardless of metric or sample size, missingness patterns C, F, H, and I have the largest bias and also the most variability of bias (though recall the variability is based on a smaller sample size due to model fitting failures). With the BI approach, regardless of metric or sample size, the bias is highest in settings with multiple missing covariates, and increases as the proportion of missingness increases, so that the highest bias occurs in settings E, F, H, and I.

**FIGURE 5:**
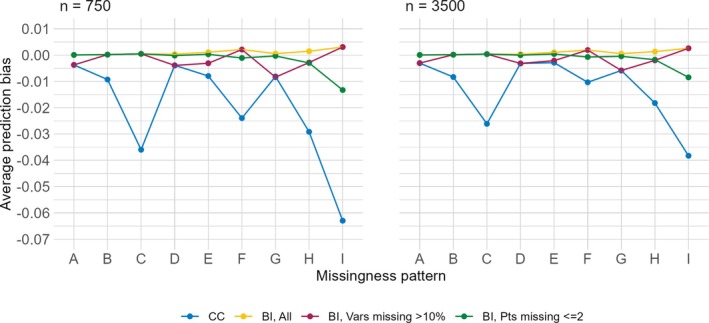
Average individual prediction bias (y‐axis) by sample size (columns) comparing the CC approach and the BI approach with three imputation methods (color) across missing data patterns (x‐axis).

A plot of the average individual prediction bias is shown in Figure 5. We find that the average individual prediction bias is quite low across all settings, and tends to be in a negative direction, indicating that the predicted risks using these missing data approaches underestimate the predicted risks in the true full data. In addition, the average individual prediction bias using the BI approach is always equal to or less than the average individual prediction bias using the CC approach. When using the BI approach, the bias is equal or lower across almost all scenarios when we impute all versus only imputing covariates missing >10% or only imputing for participants with ≤2 missing covariates. For missing data patterns A, D, E, and G, when the approach is BI, imputing only covariates missing >10% has the highest bias, whereas in missing data pattern I, imputing for participants with ≤2 missing covariates has the highest bias.

The R functions and code to run all included simulation studies are available on GitHub at https://github.com/zabore/manuscript‐code‐repository/tree/master/Mi‐Zabor_bootstrap‐impute‐predict‐tutorial.

## 8 Discussion

The development of clinical risk prediction models has proliferated in the past decade, as data sources become more available and interest in personalized medicine increases. In this article, we have provided practical guidance on the most appropriate approach to handling missing covariate data when the goal is to build a clinical risk prediction model with internal validation. Deterministic regression imputation enables predictions for future patients by excluding the outcome from the imputation models. Missing covariates for future patients can be easily imputed using regression coefficients derived from the imputation model. Therefore, the results of a clinical prediction model developed and validated in this way could easily be published for future use or deployed in an online risk prediction calculator. The step‐by‐step tutorial, coupled with a detailed discussion of the rationale behind the proposed approach, aims to guide future statisticians and clinicians when they wish to build a clinical risk prediction model but face the dual challenges of missing covariate data and a lack of external data for validation.

The results of our simulation studies reveal that bootstrapping followed by deterministic imputation, where all missing values are imputed, results in the least biased estimates of prediction model performance as measured by either the AUC or the Brier score, and in the least biased estimates of individual risk predictions. This paper did not aim to directly compare performance metrics, as that work has been done previously [13, 16]. Rather, the aim was to compare approaches to handling missing data in the context of commonly used performance metrics.

Even in settings with low levels of missing data, as low as 5% in a single covariate, we found that bootstrapping followed by imputation resulted in lower bias than complete case analysis. In settings with high levels of missing data, such as one covariate missing for 60% of participants, three covariates missing for 15%, 30% and 60% of participants, and six covariates missing in 5%, 5%, 15%, 15%, 30%, 30% or in 15%, 15%, 30%, 30%, 60%, 60%, complete case analysis was not always feasible, as the model might fail to converge, or if it did converge, it could be severely overfit. The feasibility and reliability of complete case analysis depend on the total sample size, the number of events (i.e., censoring rate), the complete case sample size, and the number of covariates included in the final prediction model. In some scenarios, the complete case dataset may be too small to even feasibly fit the model of interest. Our findings indicate that even in settings with high levels of missingness, and with a total sample size as small as 750, using bootstrapping followed by imputing all missing values resulted in relatively low bias.

There are many reasons why covariate data may be missing from datasets used to develop and internally validate a clinical risk prediction model. A common cause is the use of retrospective datasets, where variables of interest were not routinely collected but were instead captured incidentally during routine clinical care. In such cases, the missing data are likely to meet the assumption of being missing at random (MAR), meaning the probability of missingness depends only on observed variables. At other times, a single covariate may have high levels of missingness for specific reasons, such as resulting from an expensive, difficult‐to‐access, or invasive test. In such cases, the missingness may be missing not at random (MNAR), meaning it depends in part on unobserved values. Under MNAR conditions, deterministic imputation methods, such as regression imputation, may yield biased estimates [28]. Careful consideration is needed to determine whether other measured covariates could provide insight into the mechanism, particularly when MNAR is plausible. These externs extend to external validation as well, where a variable routinely collected at the institution providing the development data might not have been routinely collected at the institution providing the validation data. Reasons for missing data are complex and varied, and here we do not even address the critical issue of individuals who are entirely absent from datasets due to a lack of access to routine healthcare, which poses further threats to model fairness and applicability in real‐world settings.

In our analysis, we used the same clinical dataset and set of predictor variables as those reported in a previously published study, where variables were selected a priori by clinical experts [18]. As a result, no additional variable selection was performed during our modeling process. While this approach ensures clinical relevance, it does introduce the potential for model misspecification, which is a recognized concern in clinical risk prediction modeling. Misspecification, such as omitting important predictors, can lead to biased estimates, poor calibration, and reduced transportability of the model [14, 29]. In the context of missing data, it is also important to consider the order of variable selection and imputation. Studies suggest that variable selection should be performed after imputation to avoid underestimating variability and introducing bias [30, 31].

The development and validation of clinical risk prediction models require careful thought on many levels [32]. In this work, we have focused on one critical aspect: handling missing covariate data in the context of internal validation. It is crucial that everyone involved in developing and validating clinical risk prediction models put careful thought into the choices being made that may impact results. Above all, the primary objective should always be to develop a model that is practical and reliable for use by both patients and clinicians.

## Author Contributions

**Junhui Mi** and **Emily C. Zabor:** contributed to the formulation of the problem, the simulation studies, and drafting and editing the manuscript. **Rahul D. Tendulkar, Sarah M. C. Sittenfeld** and **Sujata Patil:** contributed to writing and editing the manuscript.

## Disclosure

The authors have nothing to report.

## Conflicts of Interest

The authors declare no conflicts of interest.

## Data Availability

The data that support the findings of this study are openly available in GitHub at https://github.com/zabore/manuscript‐code‐repository/tree/master/Mi‐Zabor_bootstrap‐impute‐predict‐tutorial.

## Appendix A Table of Simulation Settings

**TABLE A1:** Simulation scenarios.

Scenario	Missing pattern	Sample size	Imputation method
1	(A) $x_{1}$ 5%	750	All
2	(B) $x_{1}$ 15%	750	All
3	(C) $x_{1}$ 60%	750	All
4	(D) $x_{1}$ 5%, $x_{3}$ 5%, $x_{4}$ 5%	750	All
5	(E) $x_{1}$ 5%, $x_{3}$ 15%, $x_{4}$ 30%	750	All
6	(F) $x_{1}$ 15%, $x_{3}$ 30%, $x_{4}$ 60%	750	All
7	(G) $x_{1}$ 5%, $x_{3}$ 5%, $x_{4}$ 5%, $x_{7}$ 5%, $x_{10}$ 5%, $x_{11}$ 5%	750	All
8	(H) $x_{1}$ 5%, $x_{3}$ 5%, $x_{4}$ 15%, $x_{7}$ 15%, $x_{10}$ 30%, $x_{11}$ 30%	750	All
9	(I) $x_{1}$ 15%, $x_{3}$ 15%, $x_{4}$ 30%, $x_{7}$ 30%, $x_{10}$ 60%, $x_{11}$ 60%	750	All
10	(A) $x_{1}$ 5%	3 500	All
11	(B) $x_{1}$ 15%	3 500	All
12	(C) $x_{1}$ 60%	3 500	All
13	(D) $x_{1}$ 5%, $x_{3}$ 5%, $x_{4}$ 5%	3 500	All
14	(E) $x_{1}$ 5%, $x_{3}$ 15%, $x_{4}$ 30%	3 500	All
15	(F) $x_{1}$ 15%, $x_{3}$ 30%, $x_{4}$ 60%	3 500	All
16	(G) $x_{1}$ 5%, $x_{3}$ 5%, $x_{4}$ 5%, $x_{7}$ 5%, $x_{10}$ 5%, $x_{11}$ 5%	3 500	All
17	(H) $x_{1}$ 5%, $x_{3}$ 5%, $x_{4}$ 15%, $x_{7}$ 15%, $x_{10}$ 30%, $x_{11}$ 30%	3 500	All
18	(I) $x_{1}$ 15%, $x_{3}$ 15%, $x_{4}$ 30%, $x_{7}$ 30%, $x_{10}$ 60%, $x_{11}$ 60%	3 500	All
19	(A) $x_{1}$ 5%	750	Vars missing >10%
20	(B) $x_{1}$ 15%	750	Vars missing >10%
21	(C) $x_{1}$ 60%	750	Vars missing >10%
22	(D) $x_{1}$ 5%, $x_{3}$ 5%, $x_{4}$ 5%	750	Vars missing >10%
23	(E) $x_{1}$ 5%, $x_{3}$ 15%, $x_{4}$ 30%	750	Vars missing >10%
24	(F) $x_{1}$ 15%, $x_{3}$ 30%, $x_{4}$ 60%	750	Vars missing >10%
25	(G) $x_{1}$ 5%, $x_{3}$ 5%, $x_{4}$ 5%, $x_{7}$ 5%, $x_{10}$ 5%, $x_{11}$ 5%	750	Vars missing >10%
26	(H) $x_{1}$ 5%, $x_{3}$ 5%, $x_{4}$ 15%, $x_{7}$ 15%, $x_{10}$ 30%, $x_{11}$ 30%	750	Vars missing >10%
27	(I) $x_{1}$ 15%, $x_{3}$ 15%, $x_{4}$ 30%, $x_{7}$ 30%, $x_{10}$ 60%, $x_{11}$ 60%	750	Vars missing >10%
28	(A) $x_{1}$ 5%	3 500	Vars missing >10%
29	(B) $x_{1}$ 15%	3 500	Vars missing >10%
30	(C) $x_{1}$ 60%	3 500	Vars missing >10%
31	(D) $x_{1}$ 5%, $x_{3}$ 5%, $x_{4}$ 5%	3 500	Vars missing >10%
32	(E) $x_{1}$ 5%, $x_{3}$ 15%, $x_{4}$ 30%	3 500	Vars missing >10%
33	(F) $x_{1}$ 15%, $x_{3}$ 30%, $x_{4}$ 60%	3 500	Vars missing >10%
34	(G) $x_{1}$ 5%, $x_{3}$ 5%, $x_{4}$ 5%, $x_{7}$ 5%, $x_{10}$ 5%, $x_{11}$ 5%	3 500	Vars missing >10%
35	(H) $x_{1}$ 5%, $x_{3}$ 5%, $x_{4}$ 15%, $x_{7}$ 15%, $x_{10}$ 30%, $x_{11}$ 30%	3 500	Vars missing >10%
36	(I) $x_{1}$ 15%, $x_{3}$ 15%, $x_{4}$ 30%, $x_{7}$ 30%, $x_{10}$ 60%, $x_{11}$ 60%	3 500	Vars missing >10%
37	(A) $x_{1}$ 5%	750	Pts missing ≤2
38	(B) $x_{1}$ 15%	750	Pts missing ≤2
39	(C) $x_{1}$ 60%	750	Pts missing ≤2
40	(D) $x_{1}$ 5%, $x_{3}$ 5%, $x_{4}$ 5%	750	Pts missing ≤2
41	(E) $x_{1}$ 5%, $x_{3}$ 15%, $x_{4}$ 30%	750	Pts missing ≤2
42	(F) $x_{1}$ 15%, $x_{3}$ 30%, $x_{4}$ 60%	750	Pts missing ≤2
43	(G) $x_{1}$ 5%, $x_{3}$ 5%, $x_{4}$ 5%, $x_{7}$ 5%, $x_{10}$ 5%, $x_{11}$ 5%	750	Pts missing ≤2
44	(H) $x_{1}$ 5%, $x_{3}$ 5%, $x_{4}$ 15%, $x_{7}$ 15%, $x_{10}$ 30%, $x_{11}$ 30%	750	Pts missing ≤2
45	(I) $x_{1}$ 15%, $x_{3}$ 15%, $x_{4}$ 30%, $x_{7}$ 30%, $x_{10}$ 60%, $x_{11}$ 60%	750	Pts missing ≤2
46	(A) $x_{1}$ 5%	3 500	Pts missing ≤2
47	(B) $x_{1}$ 15%	3 500	Pts missing ≤2
48	(C) $x_{1}$ 60%	3 500	Pts missing ≤2
49	(D) $x_{1}$ 5%, $x_{3}$ 5%, $x_{4}$ 5%	3 500	Pts missing ≤2
50	(E) $x_{1}$ 5%, $x_{3}$ 15%, $x_{4}$ 30%	3 500	Pts missing ≤2
51	(F) $x_{1}$ 15%, $x_{3}$ 30%, $x_{4}$ 60%	3 500	Pts missing ≤2
52	(G) $x_{1}$ 5%, $x_{3}$ 5%, $x_{4}$ 5%, $x_{7}$ 5%, $x_{10}$ 5%, $x_{11}$ 5%	3 500	Pts missing ≤2
53	(H) $x_{1}$ 5%, $x_{3}$ 5%, $x_{4}$ 15%, $x_{7}$ 15%, $x_{10}$ 30%, $x_{11}$ 30%	3 500	Pts missing ≤2
54	(I) $x_{1}$ 15%, $x_{3}$ 15%, $x_{4}$ 30%, $x_{7}$ 30%, $x_{10}$ 60%, $x_{11}$ 60%	3 500	Pts missing ≤2
